# Effect of Leaves of *Caesalpinia decapetala* on Oxidative Stability of Oil-in-Water Emulsions

**DOI:** 10.3390/antiox6010019

**Published:** 2017-03-04

**Authors:** María Gabriela Gallego, Monika Skowyra, Michael H. Gordon, Nurul Aini Mohd Azman, María Pilar Almajano

**Affiliations:** 1 Chemical Engineering Department, Technical University of Catalonia, Av. Diagonal 647, 08028 Barcelona, Spain; maria.gabriela.gallego@upc.edu (M.G.G.); skowyra.monika@gmail.com (M.S.); ainiazman@gmail.com (N.A.M.A.); 2 Department of Food and Nutritional Sciences, University of Reading, Whiteknights, P.O. Box 226, Reading RG6 6AP, UK; m.h.gordon@reading.ac.uk

**Keywords:** free radicals, polyphenols, lipid oxidation, oil-in-water emulsion

## Abstract

*Caesalpinia decapetala* (Roth) Alston (Fabaceae) (CD) is used in folk medicine to prevent colds and treat bronchitis. This plant has antitumor and antioxidant activity. The antioxidant effects of an extract from *Caesalpinia decapetala* (Fabaceae) were assessed by storage of model food oil-in-water emulsions with analysis of primary and secondary oxidation products. The antioxidant capacity of the plant extract was evaluated by the diphenylpicrylhydrazyl (DPPH), Trolox equivalent antioxidant capacity (TEAC), oxygen radical absorbance capacity (ORAC) and ferric reducing antioxidant power (FRAP) assays and by electron paramagnetic resonance (EPR) spectroscopy. Lyophilized extracts of CD were added at concentrations of 0.002%, 0.02% and 0.2% into oil-in-water emulsions, which were stored for 30 days at 33 ± 1 °C, and then, oxidative stability was evaluated. The CD extract had high antioxidant activity (700 ± 70 µmol Trolox/g dry plant for the ORAC assay), mainly due to its phenolic components: gallic acid, quercetin, catechin, 4-hydroxybenzoic acid and *p*-coumaric acid. At a concentration of 0.2%, the extract significantly reduced the oxidative deterioration of oil-in-water emulsions. The results of the present study show the possibility of utilizing CD as a promising source of natural antioxidants for retarding lipid oxidation in the food and cosmetic industries.

## 1. Introduction

Oil-in-water (O/W) emulsions form the basis for many food products, such as milk, beverages, sauces and dressings. Lipid oxidation is believed to be more likely to occur in O/W emulsions due to their higher interfacial area, where the oxidative reaction is thought to be initiated [1].

The addition of antioxidants is important to prevent or retard the deterioration of the quality of lipid-based food products, which is mainly due to the attack of reactive oxygen species (ROS) [2].

Antioxidants can act as retarders by counteracting lipid oxidation in two ways: by protecting target lipids from oxidation initiators; or by hindering the propagation phase, the latter being known as chain-breaking antioxidants. This depends on various factors, such as the physicochemical characteristics of the medium and the interaction with other compounds.

Amongst the molecules considered as antioxidants, phenolic compounds are particularly important due to their high redox potentials and also because they are the most abundant antioxidants found in the diet [2]. Therefore, various phenolic antioxidants are usually incorporated into O/W emulsions to improve their oxidative stability. Synthetic antioxidants, such as butylated hydroxyanisole (BHA), butylated hydroxytoluene (BHT) and tertiary butyl hydroquinone (TBHQ), are very effective in retarding lipid oxidation [3].

Recently, there has been growing interest in the use of natural polyphenols to retard lipid oxidation due to their possible health-promoting properties and remarkable antioxidant activity [4,5]. Natural additives extracted from nuts, fruits, herbs and spices have been studied [6,7].

*Caesalpinia decapetala* (Fabaceae) (CD) has been used since ancient times in traditional Oriental medicine. It possesses antipyretic, anti-inflammatory, laxative, tonic, carminative, anticancer and antioxidant activities, amongst other [8]. Previous research on CD [9] revealed that the main chemical components were terpenoids and flavonoids, many of which have antitumor activities*.* This plant is also a rich source of tannins. The leaves of CD contain cassane diterpenoid, caesaldecan, spathulenol, 4,5-epoxy-8(14)-caryophyllene, squalene, lupeol, resveratrol, quercetin, astragalin and stigmasterol [9].

The functional properties of CD have been known and studied in various fields [10,11,12]. However, there are no studies on the effects of the addition of CD extracts into food systems. This plant may be useful in the development of new antioxidant strategies for maintaining the quality of O/W emulsions. Therefore, the aim of this study was to evaluate the antioxidant capacity and to test the oxidative stability of O/W emulsions containing ethanolic extracts of CD.

## 2. Materials and Methods

### 2.1. Plant Material

CD leaves were collected during spring (May 2016), in the District of San Martin de Porres, 123 m above sea level in Lima Province, Department of Lima, Peru. Samples were identified by the biologist Hamilton Beltran at the Museum of Natural History of the “Universidad Nacional San Marcos” (Peru) and catalogued at the “Instituto de Fitoterapia Americano”. The leaves were collected carefully, avoiding their rupture. Subsequently, the dirt and impurities were removed. The plant was identified in a research centre in Ecuador. The leaves were dried naturally at room temperature and stored in a desiccator (dessicator basic line, Labbox labware s.l., Mataró, Barcelona, Spain), which was protected from light. A representative sample (200 g) was sent to “Universitat Politècnica de Catalunya”. After reception, leaves were crushed, sieved using a sieve of 40 microns and stored inside a desiccator at room temperature until use.

### 2.2. Chemicals

Chemicals have been employed in the different methodologies of determination of the antiradical capacity of the extracts and the measurement of the antioxidant activity in the emulsions.

Reagents used in the different antiradical capacity determinations of the extracts and the measurement of the antioxidant activity in the emulsions were: 1,1-diphenyl-2-picrylhydrazyl radical (DPPH), anhydrous sodium carbonate, gallic acid, 2,2′-azino-bis (3-ethylbenzothiazoline)-6-sulfonic acid diammonium salt (ABTS), (±)-6-hydroxy-2,5,7,8-tetramethylchromane-2-carboxylic acid (Trolox), AAPH (2,2,-azobis(2-methylpropionamide)dihydrochloride, 2,4,6-tris(2-pyridyl)-*s*-triazine), catechin, epicatechin, vanillic acid purity ≥97.0%, gallic acid from Sigma-Aldrich (Alcobendas, Madrid, Spain), thiobarbituric acid, Folin-Ciocalteu reagent, methanol, hydrogen chloride, alumina (aluminium oxide), fluorescein (C_20_H_10_Na_2_O_5_), ethanol 96% *v*/*v* and ammonium thiocyanate (NH_4_SCN) from Panreac (Castellar del Vallès, Barcelona, Spain).

### 2.3. Sample Extraction

Previous studies in the laboratory have been carried out with different solvents, such as methanol 50%, pure methanol, ethanol 50%, pure ethanol, hot water, cold water and acetonitrile. The best yield of total polyphenols was obtained with 50% ethanol. Therefore, this is the solvent concentration used to make the extraction.

Dried powdered CD leaves (1.5 g) were stirred using a magnetic stirrer with a mixture of ethanol:water (25 mL 1:1 (*v/v*)) for 24 h at 4 °C; then, the mixture was centrifuged at 2500 rpm, and the supernatant was collected and stored at −80 °C. It was subsequently concentrated in a rotary evaporator (rotavapor Buchi R-210, Massó Analítica S.A., Barcelona, Spain) at 35 °C and lyophilized, protected from light. The dried extract was stored in a desiccator until use to determine the antiradical capacity.

### 2.4. Determination of Total Polyphenols and Total Flavonoid Content

Total polyphenol content was determined by colorimetric spectrophotometry following the Folin-Ciocalteu method [13]. The absorbance was measured at λ = 765 nm. The gallic acid equivalent concentration was calculated from a standard gallic acid calibration line, and the results are expressed as mg gallic acid equivalent per g of lyophilized extract.

Total flavonoid content was determined according to the procedure of Zhishen et al., (1999) [14]. Total flavonoid content was determined in triplicate. The absorbance was measured at 510 nm using a UV-4201/20 spectrophotometer (Auxilab, S.L., Navarra, Spain). Catechin was used as a standard, and total flavonoid contents were expressed as mg catechin equivalent per g of dry plant.

### 2.5. HPLC Analysis of the Extracts

Polyphenolic compounds were determined using an LC-ESI-QTOF-MS system acquired from Agilent (Wilmington, DE, USA). The LC instrument was an Agilent 1200 Series, consisting of an autosampler, two isocratic high pressure mixing pumps, a vacuum degasser unit and a chromatographic oven. The QTOF mass spectrometer was an Agilent 6520 model, furnished with a dual-spray ESI source.

Polyphenolic compounds were separated in a Zorbax Eclipse XDB C18 column (100 mm × 2 mm, 3.5 m) acquired from Agilent (Wilmington, DE, USA) and connected to a C18 (4 mm × 2 mm) guard cartridge from Phenomenex (Torrance, CA, USA). Ultrapure water (A) and acetonitrile (B), both containing 0.1% formic acid, were used as mobile phases applying the following gradient: 0–10 min, 3% B; 15–17 min, 100% B; 11 min 3% B. The mobile phase flow was 0.2 mL/min; the injection volume for standards and sample extracts was 10 µL; and the column temperature was set at 30 °C. Components in the ethanolic extract from the leaves of *C. decapetala* were identified and quantified using the reference standards: catechin, quercetin, gallic acid, 4-hydroxybenzoic acid and *p*-coumaric acid. The concentration of the extract was expressed in ng/mL.

### 2.6. Radical Scavenging Determination

We used radical scavenging assays to determine the antioxidant activity of the extract taking into account the previous studies carried out in our research group. These studies reflect that these assays are adequate to obtain reliable results [13].

Five methods were used for the evaluation of the radical scavenging activity of the extracts. The methods were performed as described by Gallego et al., (2013) [15]: the ferric reducing antioxidant power (FRAP) method, the 2,2′-azino-bis-(3-ethylbenzthiazoline)-6-sulphonic acid (ABTS^•+^) assay, the oxygen radical absorbance capacity (ORAC) assay and the 1,1-diphenyl-2-picrylhydrazyl (DPPH) method. Results were expressed in μmol of Trolox equivalent (TE) or μmol of ferulic acid equivalent per gram of plant dry weight (DW).

### 2.7. Radical Scavenging by Electron Paramagnetic Resonance Assay

Radical scavenging activity was measured by electron paramagnetic resonance (EPR) following the method of Azman et al., (2015) [16]. The extraction was executed in MeOH in 1:10 (*w*/*v*), and the soluble concentration of CD was determined as the procedure above. Results were expressed in μmol of ferulic acid equivalent per g of plant dry weight (DW).

The reaction mixture was: 100 μL of 5,5-Dimethyl-1-Pyrroline-N-Oxide (DMPO) (35 mM), 50 μL of H_2_O_2_ (10 mM), 50 μL CD extract at different concentrations, with ferulic acid (50 μL) or pure MeOH (50 μL) as the control; and 50 μL of Fe_2_SO_4_ (2 mM). These solutions were passed through a quartz tube and introduced into the EPR spectrometer (Bruker EMX-Plus 10/12, Madrid, Spain). The spectrum was recorded 10 min after the addition of the FeSO_4_ solution, when the radical adduct signal is greatest. The microwave frequency was 9.8762 GHz; microwave power was 30.27 mW; and centre field was 3522.7 Gauss (G).

### 2.8. Preparation of Stripped Sunflower Oil

Commercial sunflower oil samples were purified as described by Yoshida et al., (1993) [17] by passing them through an alumina column containing 300 g of this sorbent previously activated in an oven at 200 °C for 24 h. This step is required to remove tocopherols, peroxides and trace metals naturally occurring in sunflower oil. Purified oil was stored at −80 °C until use.

### 2.9. Preparation of Emulsions

Oil-in-water emulsions were prepared by dissolving Tween-20 (1%) in Milli-Q water and adding the purified sunflower oil (10%). The lyophilized extracts of CD were added at concentrations of 0.002%, 0.02% and 0.2% of lyophilized weight, previously reconstituted in ethanol:water (1:1 *v/v*). A negative control was prepared without extract, and two positive controls were prepared with BHA (0.004%) and Trolox (0.02%) dissolved in 1 mL of ethanol. The Tween 20 was previously mixed with water, and the oil was added dropwise continuously to this mixture, stirring with an Ultra Turrax (3000 revolutions per minute) for 10 min. The emulsion was divided into 60-mL portions. To each portion, the antioxidant sample was added, divided into triplicate aliquots of 20 mL, transferred into capped amber bottles and incubated in an oven at 33 °C in the absence of light and with constant elliptical movement. Emulsions were stored in closed vessels, the surface-to-volume ratio being 2.5. The incubation temperature chosen took into account the results obtained in previous studies in our laboratory, enabling the emulsions to be oxidized slowly without affecting the natural antioxidant [12]. The emulsions were stored for the duration of the oxidative study (31 days).

### 2.10. Determination of Peroxide Value 

The primary oxidation products formed in the above emulsions were measured periodically using aliquots of 0.005–0.1 g of each sample and determined by the ferric thiocyanate method as described by Frankel (1998), after calibrating the procedure with a series of oxidized oil samples analysed by the American Oil Chemists’ Society (AOCS) Official Method Cd 8-53 [18]. The peroxide value assay in the emulsions was performed in triplicate daily.

### 2.11. Determination of Secondary Oxidation by TBARS for Emulsions

The Thiobarbituric Acid Reactive Substances (TBARS) reagent (0.315%) for emulsions was prepared from 15% trichloroacetic acid, 0.375% thiobarbituric acid and 2.1% hydrochloric acid (37%). An aliquot of each emulsion was mixed with BHA solution, and the TBARS reagent was added. Samples were sonicated for 10 min and immersed in a water bath at 95 °C for 10 min. Then, they were centrifuged, and the absorbance of the supernatant was measured at λ = 531 nm. The results are expressed as mg malondialdehyde (MDA)/kg of emulsion. The TBARS secondary oxidation assay was evaluated once a week.

### 2.12. Headspace Volatile Analysis of Emulsions

The TBARS assay was performed in triplicate. Hexanal was measured using a TRACE GC gas chromatograph equipped with a DSQII mass spectrometer (Thermo Fisher Scientific, Madrid, Spain) with a TriPlus automatic headspace injector*.* For the preparation of the standard, an initial emulsion was prepared, placed in a vial and an amount of hexanal added, followed by stirring for 30 min. From this initial concentration, several decimal dilutions (10 dilutions) were made. A calibration line was developed with these dilutions. Emulsions (0.8 mL) in 10-mL glass vials capped with aluminium caps with PTFE/silicone septa were shaken and heated at 60 °C for 30 min in an autosampler heating block before measurement. The vapour phase (1 mL) was introduced into the chromatograph using a special syringe maintained at 65 °C. Hexanal concentrations were determined from the peak areas using a standard curve prepared from authentic hexanal. Samples were determined in triplicate.

### 2.13. Statistical Analysis

All experiments were carried out in triplicate, and mean values with standard deviations were reported. Statistical analyses were conducted using the Minitab software program (Minitab Ltd., Coventry, UK) Significant differences were identified by one-way analysis of variance (ANOVA) with Duncan’s pairwise comparison (*p* < 0.05). Statistical analysis was employed to identify significant differences between samples for all figures.

## 3. Results

### 3.1. Total Phenolic and Total Flavonoid Content

The total phenolic content of CD, expressed as mg of gallic acid per g of dry plant, was 31.58 ± 1.4 mg/g (Table 1). The total flavonoid content of CD, as, was 1.96 ± 0.01 mg of catechin per g of dry plant.

**Figure 1:**
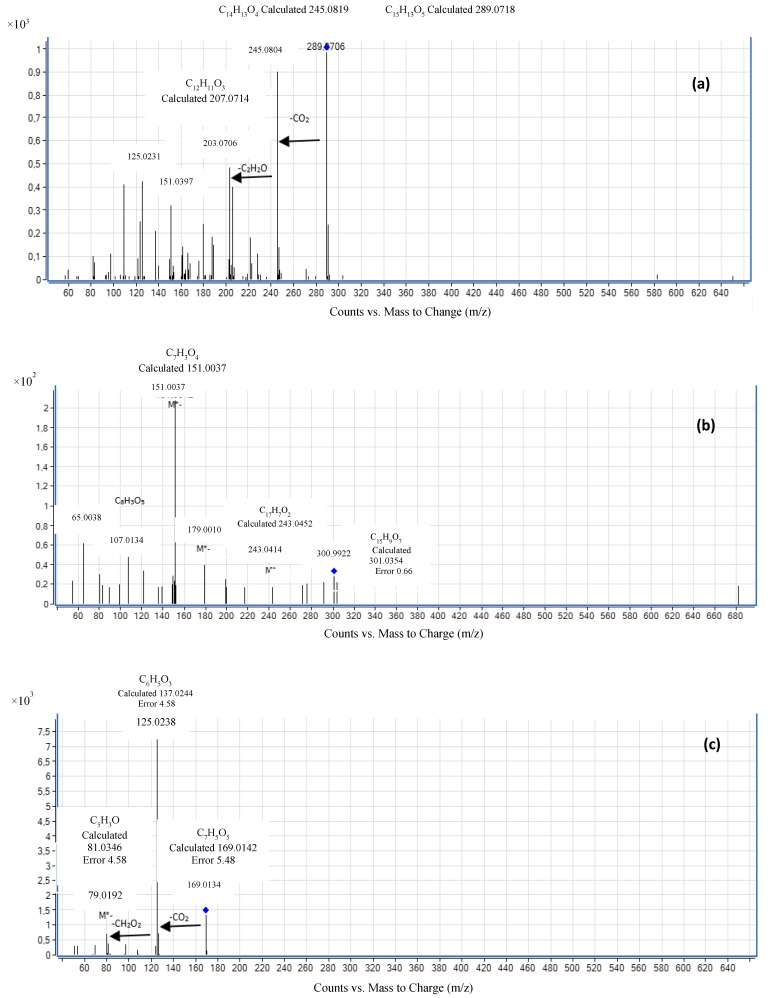

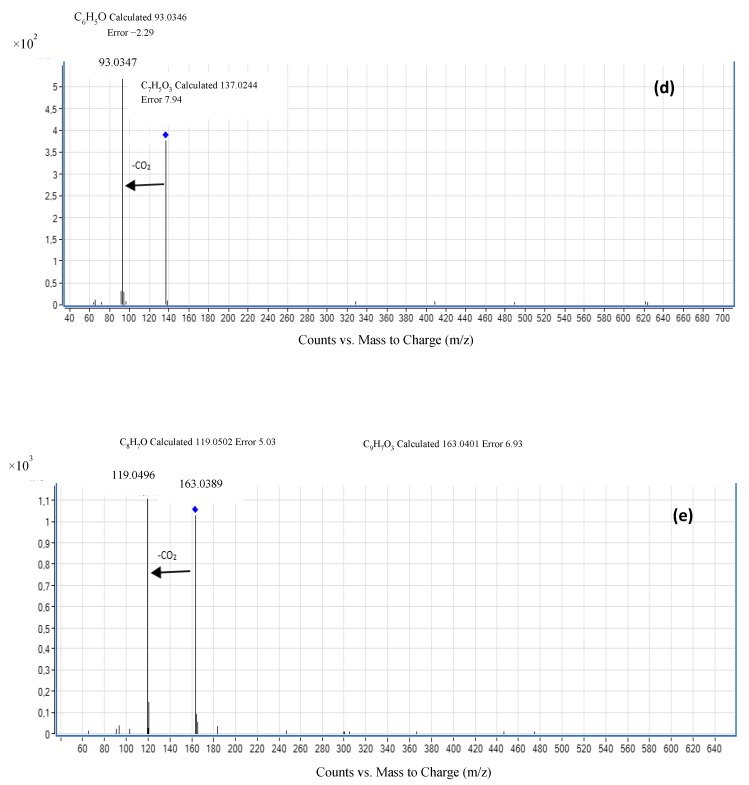
ESI (−)-MS/MS spectra corresponding to: catechin (**a**); quercetin (**b**); gallic acid (**c**); 4-hydroxybenzoic acid (**d**); and *p*-coumaric acid (**e**).

Figure 1 shows the ESI(–)-MS/MS spectra for the compounds. The analysis of the components in the CD extract showed that samples contained catechin (a), quercetin (b), gallic acid (c), 4-hydroxybenzoic acid (d) and *p*-coumaric (e). These results were obtained by comparison of spectra and retention times with reference standards. Table 2 shows the empirical formula, retention time and concentration of the compounds found in the CD extract. Gallic acid was present at the highest concentration (77,824 ng·mL^−1^), followed by catechin (669 ng·mL^−1^), *p*-coumaric acid and 4-hydroxybenzoic acid (155 ng·mL^−1^), and the lowest concentration was quercetin (64 ng·mL^−1^).

### 3.2. Radical Scavenging Activity

The radical scavenging activity can be measured by the ability of the compound to intercept free radicals. Antioxidants may act by one or several possible mechanisms, including sequestration of free radicals, hydrogen donation, metal ion chelation, electron transfer or even acting as a substrate for radicals, such as superoxide or hydroxyl.

The antioxidant capacity of CD extract was investigated by the TEAC (trolox equivalent antioxidant capacity), ORAC (oxygen radical absorbance capacity), DPPH (diphenylpicrylhydrazyl), FRAP (ferric reducing antioxidant power) and EPR (electron paramagnetic resonance) assays. Table 1 shows the values from the first four assays. The order of antioxidant activity compared to Trolox was ORAC > TEAC > DPPH > FRAP value. The antiradical activity was maintained in the range between 200 and 700 µmol Trolox/g dry plant.

### 3.3. EPR (Electron Paramagnetic Resonance) Study

The EPR radical scavenging method has been developed to evaluate the concentration of free methoxy radicals (CH3O^•^) generated in the Fenton reaction with the CD extract. The OH radical is the most reactive oxygen species found in both plant and animal cells, with a very short half-life [19].

**Figure 2:**
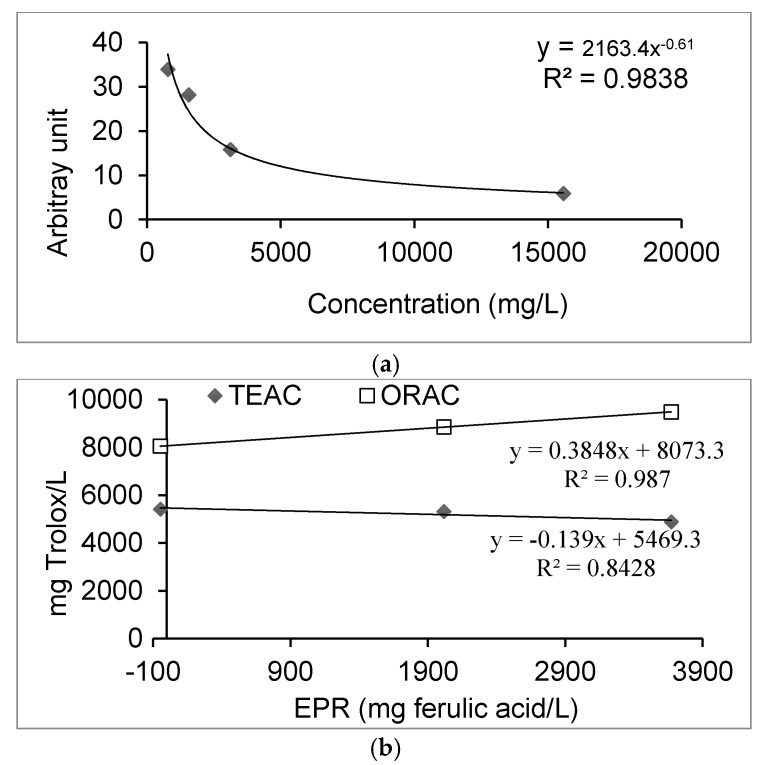
(**a**) Antioxidant activity determined by the electron paramagnetic resonance (EPR) assay for the CD (*Caesalpinia decapetala*) extract at different concentrations and (**b**) the correlation between the ORAC (oxygen radical absorbance capacity) and TEAC (trolox equivalent antioxidant capacity) assays with the EPR assay.

Figure 2 shows the decrease of arbitrary units with the increase of CD extract concentration. Due to its relatively short half-life, the methoxy radical (CH3O^•^) was identified by its ability to form a stable adduct with DMPO, the DMPO–OCH_3_. This compound can be detected by the double integration value of the EPR signal, from which the values of arbitrary units are obtained. The free radical scavenging activity of CD extracts was studied by competitive reaction in the presence of DMPO, which acted as spin trap, measured by EPR spectroscopy. The *x*-axis represents the plant extract concentration expressed as mg/L. The *y*-axis represents the arbitrary units obtained from the integrated EPR sign.

### 3.4. Effect of CD (Caesalpinia decapetala) Extract on the Oxidative Stability of Emulsions

Edible oils containing a high percentage of polyunsaturated fatty acids are very sensitive to auto- and photo-oxidation. Since autoxidation proceeds via a free radical chain reaction, antioxidants that transfer electrons and/or hydrogen atoms may retard the process. Primary oxidation was assessed by the peroxide value (PV), and secondary oxidation was assessed by the Thiobarbituric Acid (TBA) reaction and the hexanal content.

**Figure 3:**
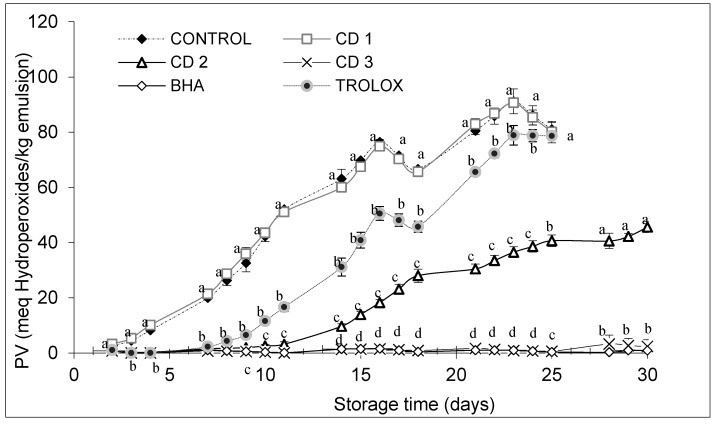
Changes in peroxide value of emulsions during storage at 33 °C for 30 days. Bars represent standard deviation (*n* = 3). Different letters in the same day (a, b, c, d) indicate significant differences between samples. PV, peroxide value; BHA, butylated hydroxyanisole.

The yield obtained from the freeze-dried CD extract was 19%. The PV of emulsion samples increased throughout storage (Figure 3).

The PV of emulsion samples containing CD at concentrations of 0.02% (CD2) or 0.2% (CD3) was lower than that of the control or sample containing Trolox (0.02%), but with 0.002% (CD1), the emulsion deteriorated at a similar rate to the control (Figure 3). The emulsion stability, assessed by hydroperoxide formation, increased with increasing concentration of the natural extract. Emulsions containing extract concentrations of 0.02% and 0.2% showed a higher stability than those prepared with the positive Trolox control (0.02%), and the 0.2% extract exhibited a similar antioxidant effect to that of BHA at 0.004%.

The PV in all of the emulsions increased during storage (30 days). At Day 1, the PV of the control and the sample containing the lowest concentration of CD extract were already significantly higher than the values for the other samples, and this shows that even in the preparation step itself, these two samples had oxidized. As the storage progressed, the PV showed a gradual increase in all of the emulsions, and the PV of the control reached 80.98 meq/kg emulsion at the end of the storage period. Emulsions containing plant extracts had PV values of 80.07 meq/kg emulsion (CD1), 48.98 meq/kg emulsion (CD2) and 2.14 meq/kg emulsion) (CD3). The PV of the emulsions containing the positive controls were 0.81 meq/kg emulsion (BHA) and 78.66 meq/kg emulsion (Trolox) at the end of the storage period.

**Figure 4:**
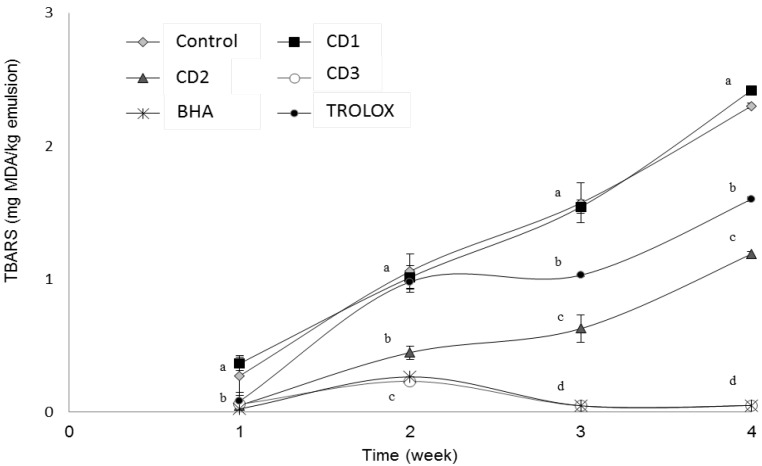
Changes in TBARS values of emulsions during the study. Bars represent standard deviation (*n* = 3). Different letters in the same day (a, b, c, d) indicate significant differences between samples.

The TBARS value was measured in the fresh and stored emulsions when PV was measured, but the samples were collected once a week. Figure 4 shows the results obtained in the TBARS test. The extracts showed an effective antioxidant activity against the formation of sequential oxidation compounds, such as malondialdehyde. As expected, the TBARS values of the control sample and the CD1 extract increased rapidly, reaching values of 2.30 and 2.41 mg malondialdehyde/kg emulsion respectively at the end of the four-week study. The CD2 extract was able to reduce lipid oxidation by 50% with respect to the control. Finally, the treatments with the CD3 extract and with BHA showed the lowest TBARS values (0.05 mg malondialdehyde/kg emulsion), indicating the greatest ability to protect the emulsions against oxidation. In this way, the order of the TBARS values (from the least effective to the most effective sample) was CD1 > control > Trolox > CD2 > CD3 > BHA at the end of the storage period.

### 3.5. Headspace Volatile Analysis

**Figure 5:**
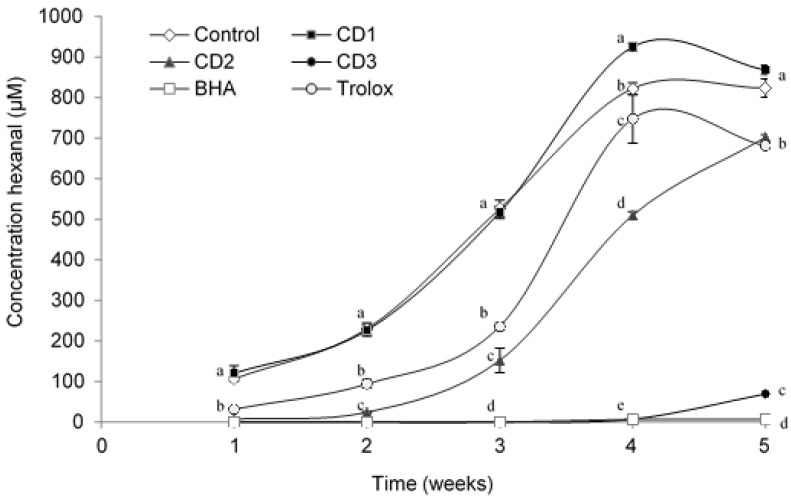
Change in the headspace hexanal concentration for oil-in-water emulsions during the study. Bars represent standard deviation (*n* = 3). Different letters in the same day (a, b, c, d, e) indicate significant differences between samples.

The concentration of volatile secondary oxidation products increases during oxidation, and hexanal is generally the main volatile produced from sunflower oil [20]. Initially in the first week, the concentration of hexanal was low in all O/W emulsion samples, and it increased gradually during the storage period (Figure 5).

Emulsions containing extracts, especially those with added lyophilized CD extract, showed, in general, lower concentrations of hexanal (702 µM for CD2 or 69.3 µM for CD3) than the negative control (without antioxidant) (823.4 µM) during the experiment. The sample containing the lowest concentration (CD1 = 0.002% of extract) had no significant antioxidant effect. Furthermore, samples containing Trolox and CD2 concentration maintained a similar behaviour until the fourth week. The behaviour of emulsions containing CD3 and BHA was similar, as was evident in Week 4 when hexanal values for CD3 (69.40 µM) increased, but samples containing additives were more stable than samples containing Trolox (681.325 µM). Both TBARS values and hexanal concentrations are measures of the secondary products of oxidation, although TBARS values include both volatile and non-volatile aldehydes. A linear correlation between TBARS values and the concentration of hexanal was found (R^2^ = 0.96%).

## 4. Discussion

### 4.1. Total Phenolic and Total Flavonoid Content

Phenolic compounds with various structures and molecular weights are formed as secondary metabolites in plants and contribute to the innate flavour of food. Flavonoids are one of the most diverse and widespread groups of natural compounds. The flavones, isoflavones, flavonols, anthocyanins and catechins are likely to be the most widespread natural phenolics [21].

The spectrophotometric quantification showed the presence of a high amount of phenolic compounds and a moderate concentration of flavonoids in the ethanol extract. The results show that the extract is rich in phenolic compounds, and these plants exhibited quite high antioxidant capacities when compared with some fruits, seeds and other plants reported in the literature [21]. Results of polyphenol content were comparable to aromatic plants, such as stem of thyme (132 ± 4.4 mg gallic acid/g dry weight) and stem (162 ± 5.2 mg gallic acid/g dry weight) and flower (52 ± 2.1 mg gallic acid/g dry weight) of lavender, reported by Gallego et al., (2013) [15]. Thyme is a rich source of polyphenols. When using LC-MS, the most intense peaks obtained correspond to free GA. The product ion at *m*/*z* 170.0215 Da was observed for this compound. This agrees with that obtained by Gallego et al., (2016) [12]. Pawar and Surana (2010) [11] reported that the concentrations of phenolic compounds in CD wood and pericarp were significant, with extraction using methanol giving a yield of 13.28 ± 0.006 mg of gallic acid per g of dry plant in the wood and 12.68 ± 0.005 mg of gallic acid per g of dry plant in the pericarp. In another study with the species *Caesalpinia digyna*, Srinivasan et al., (2007) [21] reported that extraction with methanol and water gave a yield of 44.70 ± 14.2 mg/g dry plant. The results show that the extract is rich in phenolic compounds, and these medicinal plants exhibited quite high antioxidant capacities when compared with some fruits, seeds and other medicinal plants reported in the literature [9,12].

The ethanolic extract contained several polyphenolic compounds with great antiradical activity. Authors, such as Bhat et al., (2016) [22], have also identified several active polyphenolic compounds in the ethanolic extracts of *Caesalpinia mimosoides* plant. LC-MS revealed the presence of four compounds at different retention times. The phenolic acids found (*p*-hydroxybenzoic acid and *p*-coumaric acid) showed smaller peaks under the LC-MS conditions employed. However, these were identified with reference standards. Fragments in the product ion spectrum of these compounds are shown in Figure 1, with a molecular ion at *m*/*z* 138.0317 Da for p-hydroxybenzoic acid and 164.0473 Da for *p*-coumaric acid. Pawar and Surana (2010a) [10] reported concentrations that varied from 3.93 ± 0.005 mg quercetin/g dry plant (wood of CD) to 5.26 ± 0.005 mg quercetin/g dry plant (pericarp of CD). Our analysis also revealed the presence of quercetin with a precursor ion at *m*/*z* 302.0427 Da, which confirms the findings of previous studies for species of the genus *Caesalpinia* [12]. Furthermore, it has the presence of catechin with a precursor ion *m*/*z* 290.0790 Da being demonstrated. It is evident from our analysis that the plant is rich in flavonoids.

The antioxidant activity may also be influenced by some particularly active individual phenolic compounds other than flavonoids. Many studies have revealed that phenolic contents in plants are related to their antioxidant activity, and the antioxidant activity of phenolic compounds is mainly due to their redox properties, which allow them to act as reducing agents, hydrogen donors and singlet oxygen quenchers [23]. Although Wei et al. (2013) [8] reported the presence of quercetin and rutin in CD extracts, no rutin was detected in this study.

Previous chemical analysis of this plant had revealed that the main components were terpenoids and flavonoids. Recently, the antitumor activity of the chemical constituents of CD was tested to validate the medicinal use of CD [8].

Numerous studies of the properties of species of the genus *Caesalpinia* (Fabaceae) have confirmed their effectiveness as a natural source of bioactive compounds with therapeutic applications. The genus *Caesalpinia* contains several classes of chemical compounds, including flavonoids, diterpenes and steroids. Gallic acid is an active constituent in this genus [24].

### 4.2. Radical Scavenging Activity

Radical scavenging activity was conducted by photometric determination using the ORAC, TEAC, DPPH and FRAP assays. The ORAC assay measures the loss of fluorescence (FL) of a probe (fluorescein) in the presence or absence of an antioxidant. In the presence of antioxidant, the FL decay is inhibited, and a typical ORAC assay kinetic curve is produced [25].

The ORAC value of CD was higher than those found in commonly-consumed herbs with high antioxidant capacity, including basil, marjoram, oregano, ginger, thyme and black tea (0.048 mmol/g dry plant; 0.27 mmol/g dry plant, 0.14 mmol/g dry plant, 0.39 mmol/g dry plant, 0.27 and 0.013 mmol/g dry plant, respectively) [5]. The TEAC, FRAP and DPPH assays involve an electron transfer reaction. The TEAC assay is based on monitoring the decay of the radical-cation ABTS^•+^ produced by the oxidation of 2,2′-azinobis(3-ethylbenzothiazoline-6-sulfonic acid) (ABTS) caused by the addition of a phenolic-containing sample. ABTS^•+^ has a strong absorption in the range of 600–750 nm and can be easily determined spectrophotometrically. In the absence of phenolics, ABTS^•+^ is rather stable, but it reacts energetically with a H-atom donor, such as phenolics, being converted into a non-coloured form of ABTS (Figure 2b) [26]. Our results are lower than the value reported by Gan et al. (2010) [27], who found that the antioxidant capacity of *Caesalpinia sappan* L. was 417.48 ± 10.57 µmol Trolox/g dry plant, although the results are of similar magnitude, and natural variability occurs due to growing conditions, etc. According to the literature, the genus *Caesalpinia* provides strong antioxidant activity, which has been confirmed [10,21,28,29]. In addition, the antioxidant capacity was tested using the “stable” free radical DPPH. This otherwise stable free radical is reduced from violet to yellow in the presence of antioxidants, and the change can be monitored spectrophotometrically [30]. The extract gave a relatively low percentage inhibition, with values of 39.8%. The percentage inhibition during the 75 min of the experiment did not reach stability, and components continued to react with the radical after this time. Hence, the low DPPH value is likely to be partly due to the steric inhibition of the reaction between the bulky antioxidants and the DPPH radical, which has two bulky aromatic groups attached to the nitrogen radical. Bhat et al., (2016) have demonstrated the strong activity against DPPH in studying *Caesalpinia mimosoides*.

The amount of plant extract needed to decrease the initial DPPH^•^ concentration by 50% (IC_50_) is a parameter widely used to measure the antioxidant activity. The lower the IC_50_, the higher the antioxidant power is. Our result (1 mg/mL) was similar to the value reported by Muñoz-Ortiz et al. (2011) [31], who found an IC_50_ of 1.30 mg/mL for leaves of *Caesalpinia pluviosa*, which were collected in Ballivian Province and were deposited in the National Herbarium of Bolivia in La Paz (number of vouchers: VM6). However, the IC_50_ value for *Caesalpinia bonducella* was much smaller at IC_50_ = 74.73 μg/mL [32], as for the bark of *C. pyramidalis* IC_50_ = 16.98 ± 1.34 μg/mL, which was incorporated in the Herbarium UFP Geraldo Mariz, Department of Botany, Federal University of Penambuco, with the number 60.195 [33], indicating more active antioxidants in the bark of this plant. The FRAP value is a measure of the capacity of the antioxidant to reduce ferric (III) to ferrous (II) ions. The FRAP assay also takes advantage of electron-transfer reactions. In this assay, a ferric salt, Fe (III)(TPTZ)_2_Cl_3_ (TPTZ) 2,4,6-tripyridyls-triazine), is used as an oxidant. The redox potential of the Fe (III) salt (~0.70 V) is comparable to that of ABTS^•+^ (0.68 V). Therefore, essentially, there is not much difference between the principles of the TEAC assay and the FRAP assay, except the former is carried out at neutral pH and the latter under acidic (pH 3.6) conditions [34].

The antioxidant capacity measured by this assay was lower than that measured by the other assays (200 ± 10.0 µmol Trolox equivalent/g dry plant). This value is lower than the values reported for *Caesalpinia sappan* of 313.50 ± 44.66 µmol and 0.324 mmol Trolox equivalent/g dry plant [27]. However, the FRAP value reported by Gan et al., (2010) [27] was in the whole plant, whereas our result is specific to the leaves of the medicinal plant CD.

The radical-scavenging activity of phenolic acids depends partly on the number of electron donor hydroxyl and methoxy substituents, which increase the stability of the phenoxy radicals. Gallic acid with three hydroxyl groups and a carboxyl group is very active in reducing free radicals [35].

According to the literature, the medicinal plant species of *Caesalpinia* have many biological activities. For example, *C. sappan* showed antibacterial activity and had the potential to be developed into an antibiotic. Plants of this species are often used for the prevention and treatment of cardiovascular and cerebrovascular diseases, because they improve blood circulation or stop bleeding [36]. Because of their high antioxidant capacities, it is possible that these plants will be beneficial for cardiovascular and cerebrovascular diseases caused by oxidative stress, and they might be developed into a functional food or drug in the future. However, further evidence relating to their bioavailability and bioactivity is required.

### 4.3. EPR Study

Electron paramagnetic resonance (EPR) spin trapping is an established technique for identifying and quantifying free radicals. The use of the EPR technique in food analysis studies is very common [37]. The CD extract at different concentrations competes with the spin trap in the scavenging of methoxy radicals. Thus, the effect reduces the amount of radical adducts and, accordingly, reduces the intensity of the EPR signal. The graph indicates that the area of the signal of the spectrum decreased as the amount of CD increased. This study confirmed that the scavenging activity of the CD extracts containing polyphenol constituents could be measured by the decrease of the intensity of the spectral bands of the adduct DMPO–OCH_3_ in the EPR spectrum as the amount of antioxidant was increased. The best fit with the EPR signal intensity was shown as an exponential function, where it corresponds to the following equation (expressed in g/L):

*Y* = 2163.4 *x*^−0.61^, *R^2^* = 0.9838

In the figure, it can be seen that the exponential value of the spectrum signal decreased as the concentration of the CD extract increased. Likewise, a good correlation was observed with the anti-radiations tests, ORAC and TEAC, being 99% and 84%, respectively. With this study, it was stated that the scavenging activity of the extract can be measured by the decrease in the intensity of the formed adduct bands (DMPO–OCH_3_) in the EPR spectrum. Similar results have been obtained by Aini et al., (2015) [16] in their EPR study with the extract from the plant *Convolvulus arvensis*, which exhibited a strong scavenging activity against the methoxy radical produced by the Fenton reaction. In another study with *Quillaja* saponin extract, an antioxidant effect was shown by the EPR test, with the extract showing antioxidant activity towards hydrophilic and hydrophobic radicals [38].

### 4.4. Effect of CD Extract on Oxidative Stability of Emulsions

In order to avoid the physical effects on lipid oxidation of the samples, homogenization of the emulsions was performed according to previous studies to maintain a suitable small droplet size to avoid cream formation.

The protective effect of plant extracts on lipid oxidation in oil-in-water systems is a consequence of the presence of active phenolic compounds in the extract. Several research groups have determined the antioxidant and protective effects of herbs on fats and oils. Plants of the Labiatae family contain active antioxidants with rosemary being a very good source, although oregano, sage and thyme also have good antioxidant capacities [39]. Rosemary extracts are relatively effective in oils, but much less effective in emulsions due to the polar nature of the antioxidants. Oregano extract was also more active in oil than emulsion, although sage extract was relatively effective in both media [40].

The control sample reached the highest hydroperoxide/kg emulsion content at the end of the experiment. Comparing results for Day 25, it was observed that the control reached 80 meq hydroperoxides/kg emulsion. Emulsions containing CD1 and Trolox showed a very similar deterioration. The emulsion with CD2 was able to reduce hydroperoxide formation by half (40 meq hydroperoxides/kg emulsion), and the CD3 extract and the synthetic antioxidant BHA were the most effective samples in retarding the oxidative stability of the emulsions, reaching PV values after 25 days of 0.61 and 0.37 meq hydroperoxides/kg of emulsion, respectively.

According to the Codex Alimentarius, the maximum acceptable level of PV for vegetable oils is 10 milliequivalents of O_2_/kg of oil. Emulsions corresponding to the control sample and CD1 extract exceeded this limit after the four-day study. However, the emulsion with the addition of CD3 at Day 25 remained still well below this value. Other studies with an extract from the tara plant of the same genus also gave very promising results, with the formation of hydroperoxides after eight days of storage at 38 °C being below 20 meq hydroperoxides/kg emulsion [41]. Antioxidants can scavenge free radicals at the oil-water interface, creating a protective barrier and preventing radicals from entering the organic phase and accelerating lipid oxidation [42].

In our previous studies with this plant in O/W emulsions, we observed the changes in polyphenolic compounds over the course of 20 days [12]. Certain polyphenolic compounds are stable, and others undergo polymerization reactions, epimerization or suffer a loss over time. Quercetin was one of the compounds that increased over time, just as *p*-coumaric acid also experienced a considerable rise in the first 10 days. It can be said that this plant contains bioactive compounds that can contribute to the protection of the emulsions against oxidation. Malondialdehyde and other TBA reactive substances are produced as a result of the oxidation of polyunsaturated fatty acids. According to the analysis, after 30 days, the TBA values of emulsion samples containing added plant extracts at >0.02% were lower than that of the control, with the order of activity of additives similar to that determined by the PV. No significant differences were found between the TBARS values for emulsions containing BHA and CD3 antioxidants (*p* < 0.05). In previous studies of this plant in meat products, we have obtained very satisfactory results, protecting the meat against lipid oxidation. At a concentration of 0.5% of *C. decapetala*, the formation of TBARS was reduced to 1.7 mg malondialdehyde/kg sample after 11 days of study, which confirms the effectiveness of this plant as a source of natural antioxidants in spite of this being a very different food model [43]. These results were lower than those reported by Gallego et al., (2013) [15] for plants of the Lamiaceae family, where studies of rosemary and thyme leaves at 100 ppm concentration in O/W emulsions showed that thyme leaves reduced the formation of TBARS to 2.50 mg malondialdehyde/kg emulsion after three weeks and rosemary leaves 1.79 mg malondialdehyde/kg emulsion.

### 4.5. Headspace Volatile Analysis

Aldehydes are the volatile compounds most frequently produced in the secondary oxidation of lipids. These generate unpleasant tastes and odours. One is hexanal, a sensitive indicator in lipid oxidation [43].

The results showed that emulsions containing extracts from CD have good oxidative stability during storage, which can be attributed to the antioxidant activity of the phenolic compounds in the herb, including phenolic acids, and the flavonoids catechin and epicatechin. A good proportion of gallic acid is present at the interface in oil in water emulsions stabilized by Tween 20. Gomes et al., (2016) [44] in their study noted that O/W emulsions showed higher gallic acid retention during the storage time. Therefore, the antioxidant is placed at the correct location (in the interface) to react with free radicals generated in the aqueous phase. Less polar flavonoids, such as quercetin, would also concentrate at the interface and make important contributions to the antioxidant capacity [45]. The results show that CD phenolics were effective in inhibiting the formation of hexanal in the oil-in-water emulsions. Similar results have been obtained by Gallego et al., (2015) [43] in a study of the extract of CD as a lipid oxidation inhibitor in beef hamburgers, finding a concentration of 0.5% of CD values around 6 ppm of hexanal well below the control (9 ppm). This is important because in order for an antioxidant to be effective, it must be able to inhibit the formation of volatile secondary lipid oxidation products that are perceived as off-flavours.

## 5. Conclusions

The results of this study show that ethanolic CD extracts exerted a significant effect on the stability of oil-in-water emulsions, especially at a 0.2% concentration, were more effective than Trolox (0.02%) and comparable in activity to BHA (0.004%). The extract had strong antioxidant activity and was rich in polyphenols. The presence of gallic acid, catechin, epicatechin and vanillic acid contributed significantly to the antioxidant activity of the extract.

Consumer demand for healthy food products provides an opportunity to develop antioxidants as new functional foods. The discovery of the great antioxidant activity of CD extracts in O/W emulsions draws interest to their use as an alternative to synthetic preservatives for the food industry, especially as an antioxidant for the conservation of fats.

## Figures and Tables

**Table 1:** Total polyphenol content and antioxidant activity assessed by the ORAC (oxygen radical absorbance capacity), TEAC (trolox equivalent antioxidant capacity), FRAP (ferric reducing antioxidant power) and DPPH (diphenylpicrylhydrazyl) assays for the studied extract.

Different Methods and Units	Value
Total polyphenols (mg gallic acid/g dry plant)	31.58 * (1.40)
Flavonoids (mg catechin/g dry plant)	1.96 * (0.01)
TEAC (µmol Trolox/g dry plant)	360 * (10)
ORAC (µmol Trolox/g dry plant)	700 * (70)
FRAP (µmol Trolox/g dry plant)	200 * (10)
DPPH (µmol Trolox/g dry plant)	300 * (20)

* Mean values of three replicates (*n* = 3); standard deviations are included in parenthesis.

**Table 2:** Polyphenols identified by HPLC-MS in *Caesalpinia decapetala* (CD) extract.

Name	Formula	Monoisotopic Molecular Weight (Da)	Retention Time (min)	Concentration (ng·mL^−1^)	S.D.
Catechin	C_15_H_14_O_6_	290.0790	11.6	669	(7)
Quercetin	C_15_H_10_O_7_	302.0427	16.8	64	(5)
Gallic acid	C_7_H_6_O_5_	170.0215	3.1	77,824	(40)
4-hydroxybenzoic acid	C_7_H_6_O_3_	138.0317	10.8	155	(10)
*p*-coumaric acid	C_9_H_8_O_3_	164.0473	13.5	155	(9)

Mean values of three replicates for monoisotopic molecular weight, retention time and concentration values of three replicates (*n* = 3); S.D.: standard deviations are included in brackets.
